# Supplementary material for: Effectiveness and Safety of Ayurvedic Medicines in Type 2 Diabetes Mellitus Management: A Systematic Review and Meta-Analysis
Source: Front Pharmacol. 2022 Jun 8;13:821810. doi: 10.3389/fphar.2022.821810 (PMC9213670; doi:10.3389/fphar.2022.821810)
Supplement: Supplementary file 1 [file DataSheet1.zip › Supplementary Material/Forest and Funnel Plots/Enicostemma axillare (versus OAD)/Enicostemma axillare.pdf]

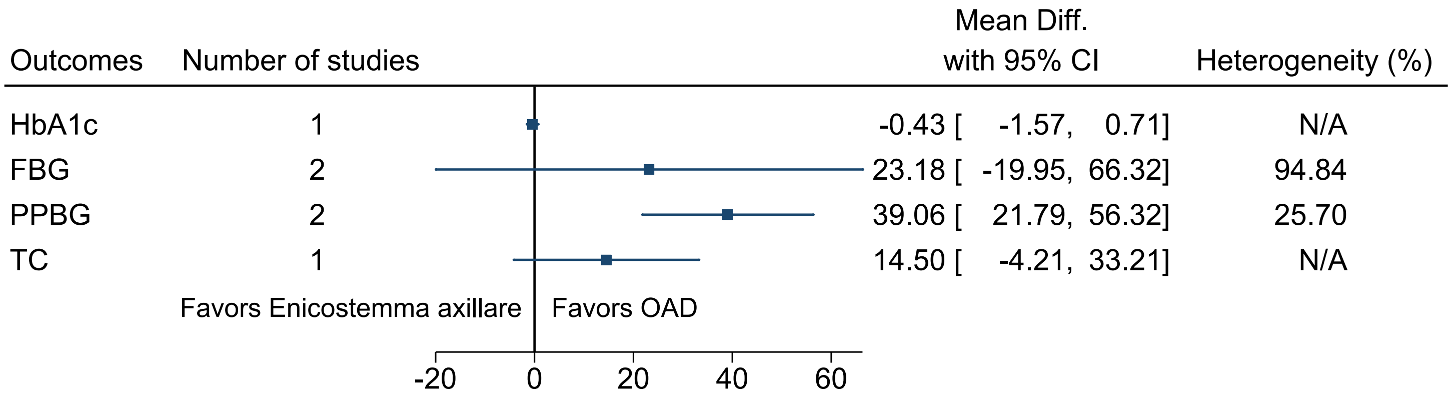
Outcomes
Number of studies
Mean Diff. with 95% CI
Heterogeneity (%)
HbA1c
1
-0.43 [-1.57, 0.71]
N/A
FBG
2
23.18 [-19.95, 66.32]
94.84
PPBG
2
39.06 [21.79, 56.32]
25.70
TC
1
14.50 [-4.21, 33.21]
N/A
Favors Enicostemma axillare
Favors OAD
-20
0
20
40
60
